# A multi‐centre cohort study investigating the outcome of synovial contamination or sepsis of the calcaneal bursae in horses treated by endoscopic lavage and debridement

**DOI:** 10.1111/evj.13180

**Published:** 2019-10-16

**Authors:** C. M. Isgren, S. E. Salem, E. R. Singer, C. E. Wylie, G. Lipreri, R. J. T. Y. Graham, B. Bladon, J. C. Boswell, A. R. Fiske‐Jackson, T. S. Mair, L. M. Rubio‐Martínez

**Affiliations:** ^1^ Department of Equine Clinical Science Institute of Veterinary Science University of Liverpool Wirral UK; ^2^ Department of Epidemiology and Population Health Institute of Infection and Global Health University of Liverpool Wirral UK; ^3^ Department of Surgery Faculty of Veterinary Medicine Zagazig University Zagazig Egypt; ^4^ Rossdales Equine Hospital Exning, Newmarket Suffolk UK; ^5^ The Royal (Dick) School of Veterinary Studies and Roslin Institute The University of Edinburgh Midlothian UK; ^6^ Donnington Grove Veterinary Group Newbury Oxfordshire UK; ^7^ The Liphook Equine Hospital Forest Mere Liphook Hampshire UK; ^8^ Equine Referral Hospital Royal Veterinary College North Mymms Hertfordshire UK; ^9^ Bell Equine Veterinary Clinic Mereworth, Maidstone Kent UK; ^10^ Sussex Equine Hospital West Sussex UK

**Keywords:** horse, infection, tendon injury, bursoscopy, bacterial isolates, antimicrobials

## Abstract

**Background:**

Previous studies investigating factors associated with survival following endoscopic treatment of contamination/sepsis of the calcaneal bursa are limited.

**Objectives:**

To investigate the factors associated with survival in horses with contamination/sepsis of the calcaneal bursae treated endoscopically and to describe the bacterial isolates involved in the synovial infections.

**Study design:**

Retrospective analysis of clinical records.

**Methods:**

Medical records from 128 horses with contamination/sepsis of the calcaneal bursae treated by endoscopic lavage at seven equine hospitals were reviewed. A follow‐up questionnaire was used to determine survival and return to athletic performance. Descriptive statistics and Cox proportional hazards survival models were used to determine factors associated with survival.

**Results:**

Horses underwent one (n = 107), two (n = 19), or three (n = 2) surgeries. Survival to hospital discharge was 84.4%. Univariable survival analysis revealed that administration of systemic antimicrobials prior to referral was associated with reduced mortality (hazard ratio, [HR] 0.41, 95% CI 0.18–0.91, P = 0.03). Increased mortality was associated with bone fracture/osteomyelitis (HR 2.43, 95% CI 1.12–5.26, P = 0.03), tendon involvement (≥30% cross sectional area) (HR 3.78 95% CI 1.78–8.04, P = 0.001), duration of general anaesthesia (HR 1.01, 95% CI 1.00–1.02, P = 0.04), post‐operative synoviocentesis (HR 3.18, 95% CI 1.36–7.43, P = 0.006) and post‐operative wound dehiscence (HR 2.5, 95% CI 1.08–5.65, P = 0.04). Multivariable Cox proportional hazards model revealed reduced mortality after systemic antimicrobial administration prior to referral (HR 0.25, 95% CI 0.11–0.60, P = 0.002) and increased mortality with tendinous involvement (≥30% cross‐sectional area) (HR 7.92, 95% CI 3.31–19.92, P<0.001). At follow‐up (median 30 months, range 0.25–13 years, n = 70) 87.1% horses were alive, 7.1% had been euthanised due to the calcaneal injury and 5.7% had been euthanised for unrelated reasons. From 57 horses with athletic performance follow‐up, 91.2% returned to the same/higher level of exercise, 5.3% to a lower level and 3.5% were retired due to persistent lameness of the affected limb.

**Main limitations:**

Retrospective study and incomplete follow‐up.

**Conclusion:**

Endoscopic treatment of contamination/sepsis of the calcaneal bursae has an 84% survival rate to hospital discharge. Tendinous involvement reduced survival whilst systemic antimicrobials administration prior to referral improved survival.

## Introduction

Infection or contamination of the calcaneal bursae is a common sequela of wounds to the plantar aspect of the tarsus. The calcaneal bursae are a collection of synovial structures associated with the *tuber calcanei* (TC) including: the acquired *bursa subcutanea calcanea* (SCB), located superficial to the superficial digital flexor tendon (SDFT); the intertendinous *bursa calcanea subtendinea musculi flexoris digit. superficialis*, situated between the SDFT and gastrocnemius tendon (GNT); and the *bursa tendinis calcanei*, which lies dorsal to the insertion of the GNT on the TC 1, 2, 3, 4. These latter two bursae are continuous 5, and an anatomical study using latex injections demonstrated 100% communication medially with 50% communicating on the lateral aspect 6, although conflictingly the opposite anatomical communications have been reported endoscopically 7. Thus, the term calcaneal bursa will be used to refer to these two bursae, with the acronym SCB for the *b.subcutanea calcanea* or subcutaneous calcaneal bursa. Injuries to the plantar aspect of the tarsus may also involve the plantar ligament (PL), distal plantar ligament, long plantar ligament, plantar fascia, tarsal sheath and/or tarsocrural joint. The high motion in this area can lead to difficulty achieving wound closure, with wound breakdown commonly occurring 8. As a result, patients often require prolonged hospitalisation.

Endoscopic treatment under general anaesthesia is considered the gold standard for synovial infections with survival rate of 86% to hospital discharge 9. The only previous report investigating the outcome of horses with calcaneal bursae contamination/sepsis included a mixture of conservative and surgical treatments, primarily debridement and through‐and‐through lavage, with only one case receiving endoscopic treatment 10. In that study, 75% of horses with sepsis of the calcaneal bursa survived to hospital discharge. The poorest outcome (44% survival) was for horses with sepsis of the calcaneal bursa with radiographic abnormalities of the TC. Knowledge of the risk factors associated with poor outcome in horses with calcaneal bursae contamination/sepsis that undergo endoscopic treatment is warranted to improve outcomes and to advise owners during decision‐making.

The aims of this study were to 1) analyse the outcome of horses with contamination/sepsis of the calcaneal bursae following endoscopic treatment, 2) identify prognostic factors associated with survival and 3) describe the bacterial isolates involved in the synovial infections. We hypothesised that endoscopic treatment of synovial contamination/sepsis of the calcaneal bursa carries a favourable outcome and that bone or tendinous involvement negatively affects outcome.

## Materials and methods

### Study design and case selection

The case records of horses that presented with synovial contamination/sepsis of the calcaneal bursae to seven equine referral hospitals in the UK (University of Liverpool Equine Hospital, Rossdales Equine Hospital, Bell Equine Veterinary Clinic, Royal Veterinary College, University of Edinburgh, Liphook Equine Hospital and Donnington Grove Equine Hospital) between the years 2002 and 2016 were reviewed. Inclusion criteria for the study were horses with synovial contamination/sepsis of the calcaneal bursae and treatment with endoscopic lavage and debridement under general anaesthesia. Synovial contamination or sepsis was diagnosed when ≥1 of the following criteria were met: elevation of all synovial fluid parameters (total nucleated cell count [TNCC] ≥10 × 10^9^ cells/l, >80% polymorphonuclear cells, total protein >30 g/l); direct communication of a wound with the synovial cavity confirmed by distension with saline or endoscopically; or positive bacteriological culture and/or visualisation of intracellular bacteria on a cytological smear 9. Tendon/ligament injury (SDFT/GNT/PL) or bone injury (TC) were diagnosed based on radiographic, ultrasonographic or endoscopic findings. Tendon/ligament injuries were graded initially according to the degree of damage (none, <30%, 30–50%, >50% of cross‐sectional area [but incomplete] and complete), and were later re‐categorised as <30% or ≥30% of total cross‐sectional area for statistical purposes. Moderate/severe tendon/ligament injury was defined as ≥30% of total cross‐sectional area affected. *Tuber calcanei* pathology was graded according to the degree and nature of damage (none, minor fragmentation, presence of a fracture and/or osteomyelitis). Moderate/severe bone damage was defined as presence of a fracture and/or osteomyelitis and excluded cases with only minor fragmentation. All surgeries were performed by ECVS/ACVS diplomats or surgeons who had completed a surgical residency but not yet sat the diploma exams. Standard technique of calcaneal bursoscopy was used 7.

### Data collection and follow‐up

Data were collected for pre‐, intra‐ and post‐operative variables considered a priori as risk factors for poor survival for synovial infections and orthopaedic injuries (Supplementary Item 1). Preoperative variables included: participating hospital, age, sex, breed, use, time to referral, affected limb, lameness grade on hospital admission, presence of an open wound, wound location, systemic antimicrobials administered prior to referral, local antimicrobials administered prior to referral, intrasynovial antimicrobials administered on hospital admission and results of microbiological culture. Intraoperative data included: duration of general anaesthesia, whether synovectomy was performed during surgery, local antimicrobials administered during surgery, intravenous regional perfusion of antimicrobials administered during hospitalisation, SCB involvement only, calcaneal bursa involvement only, involvement of both bursae, any bone damage, moderate/severe bone damage (fracture/osteomyelitis), any tendon lesion, moderate/severe tendon damage (≥30% cross sectional area) and if primary wound closure was achieved at the end of the surgery. Post‐operative data collected included: limb immobilisation after surgery with bandage, cast bandage or cast (separate variables), post‐operative synoviocentesis, total number of endoscopic lavages, post‐operative wound dehiscence and duration of hospitalisation. Follow‐up was obtained using a structured telephone questionnaire with owners/trainers/referring veterinarians and online performance records for racehorses. The questionnaire used during the telephone interviews (Supplementary Item 2) contained information about whether further treatment was required following discharge, the level of soundness achieved and whether the horse returned to lower, same or higher level of athletic function compared with before the injury. At five hospitals data collection and follow‐up were obtained by the first author (C.M.I.) and at the remaining two hospitals it was performed by one of the co‐authors (R.J.T.Y.G. and C.E.W.).

### Data analysis

All variables recorded were screened for missing or outlying data points and, when possible, the case electronic records were re‐examined to confirm if these observations were accurately recorded. Descriptive statistics were generated for all variables in the data set. Categorical variables containing a small number of observations in some of the categories were condensed into fewer biologically plausible groups (e.g. breed was categorised as group 1 (Warmblood, Warmblood cross, Thoroughbred, Thoroughbred cross, Standardbred, Lusitano and Arab) and group 2 (pony, cob and draught) and sex was re‐categorised into male (stallions and geldings) and female 11. Survival time was measured as time from the date of surgery to the date of death or censoring. Horses were censored if they were lost to follow‐up or if they were still alive at the end of the study. Thirty‐eight horses in the study did not have data recorded for follow‐up survival and were censored at the time of hospital discharge. The cumulative probabilities of survival were explored using Kaplan–Meier plots.

The association between the probability of survival and predictor variables was investigated using the Cox proportional hazards models. In this analysis, variables containing >25% missing observations were excluded to avoid misleading results due to the small sample size and the fewer number of events (deaths) recorded during the follow‐up period. Variables that showed some evidence of association with probability of survival (likelihood ratio test P<0.25) at univariable analysis were considered to build a Cox proportional hazards multivariable model. Collinearity between continuous variables was investigated using Pearson’s correlation coefficient and between variables retained in the final multivariable model using variance inflation factors (VIF) 12. The functional form of the relationships between continuous explanatory variables and the probability of survival were investigated using penalised regression models 13. A forward stepwise selection approach was used in model building where variables were added in succession and retained if they resulted in improved model fit as indicated by the likelihood ratio test for model comparison. Excluded variables were sequentially added back to the final model to make sure no important or confounding variables were excluded. The effect of hospital on survival was also tested as a frailty term (a random‐effect) in the final model. The proportional hazards assumption of the Cox regression model was evaluated for variables retained in the final model using the Therneau‐Grambsch non‐proportionality test 14 and influential data points were investigated in a delta‐beta plot. Statistical analyses were performed in R version 3.3.0 15 and the ‘survival’ statistical package version 2.39.4 was used to perform survival analysis 16. Critical probability was set at <0.05 for all analyses.

## Results

### Descriptive statistics and hospital survival

**Figure 1:**
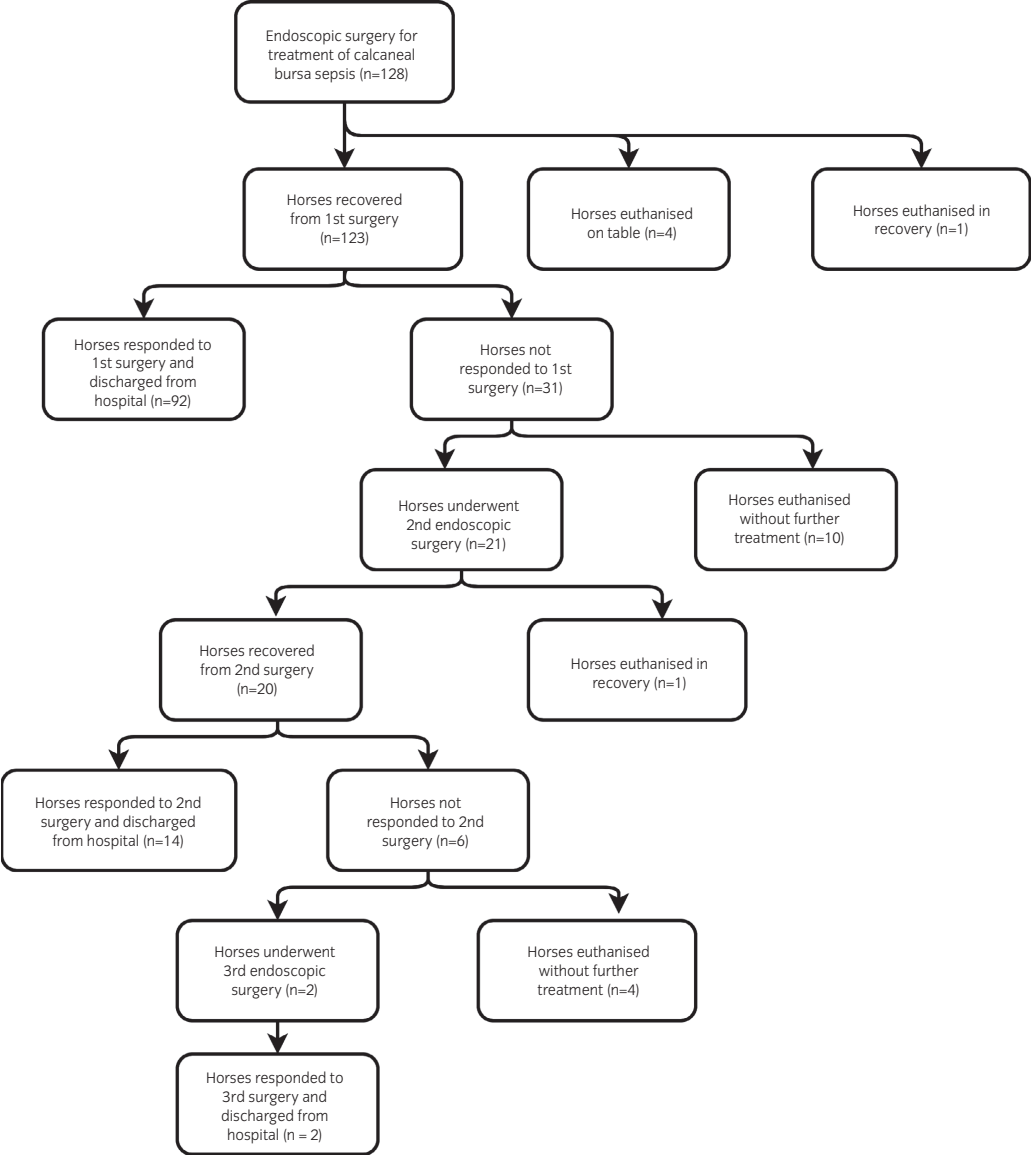
Flow chart to show recruitment of horses into, and their progression through the study to hospital discharge. ‘Responded’ = clinical signs of resolution of infection. ‘Not responded’ ＝clinical signs of persistent infection of calcaneal bursa.

The study included 128 horses (75 female, 53 male, age range 3 months to 26 years [median 9 years]). Median time to referral was 1.5 days (range 0–7 days). Survival to hospital discharge was 84.4% (108/128 horses). Four horses were euthanised during surgery due to the extent of the calcaneal injury but were included in the survival analysis. One horse was euthanised following a catastrophic fracture of the contralateral tibia during recovery from general anaesthesia after the first surgery and this horse was excluded from the survival analysis. Of 123 horses that recovered from anaesthesia, 108 (87.8%) horses survived to discharge from the hospital. A flow chart to show recruitment of horses into, and their progression through the study to hospital discharge is shown in Figure 1. The clinical signs of 92 (74.8%) horses improved following the first surgery while 31 (24.2%) horses showed persistent lameness and elevated synovial fluid markers consistent with synovial sepsis as outlined in the methods. Ten of these horses were euthanised without a second surgery, while 21 horses underwent a second endoscopic surgery. A second horse was euthanised during recovery from the second general anaesthesia due to a catastrophic tibial fracture of the contralateral limb. This horse was included in the analysis to the point of euthanasia, as the first surgery had not adequately cleared the infection. Twenty horses recovered from general anaesthesia from the second surgery and 14 were later discharged from the hospital, while six horses failed to respond to treatment including persistent lameness and elevated synovial fluid markers consistent with synovial sepsis as outlined in the methods. Of these six horses, four horses were euthanised due to signs of persistent synovial contamination/sepsis while two horses underwent a third endoscopic surgery and were later discharged. Overall, one surgery was performed in 107, two surgeries in 19 and three surgeries in 2 horses.

Both the calcaneal bursa and the SCB were affected in 42.5% (n = 54/127) of horses while in 48.0 % (n = 61/127) only the calcaneal bursa was affected and in 9.4% (n = 12/127) only the SCB was affected. All horses survived when only the subcutaneous bursa was affected therefore statistical analysis was not possible. There was bone involvement (fragmentation, fracture, osteomyelitis) of the TC in 23.6% (n = 30/127) of horses and of these 14 horses had contamination/sepsis of both the CB and SCB while the remaining 16 horses had involvement of only the CB. The bone injury was graded as moderate/severe in 19.7% (n = 25/127) (excluding fragmentation) and of these nine cases had contamination/sepsis of both the CB and the SCB while the remaining 16 horses involved only the CB. There was injury of the SDFT/GNT/PL, including mild fibrillation, present in 58.3% (n = 74/127) of horses. Tendon injury affecting ≥30% cross‐sectional area (moderate/severe injury) was present in 20.5% (n = 26/127) of horses. PL injury was limited to <30% cross‐sectional area in all cases and therefore there were no PL injuries in the moderate to severe group. Moderate/severe tendon injury of both the SDFT and GNT was present in 5.5% (n = 7/127) horses. A bacteriological culture was submitted in 32.3% (n = 41/127) and 61.0% (n = 25/41) had a positive culture. The most common bacteria isolated were *Staphylococcus* spp. (36.0%, n = 9/25) followed by *Streptococcus* spp. (28.0%, n = 7/25) and *Escherichia coli* (20.0%, n = 5/25).

### Follow‐up survival and return to athletic function

**Figure 2:**
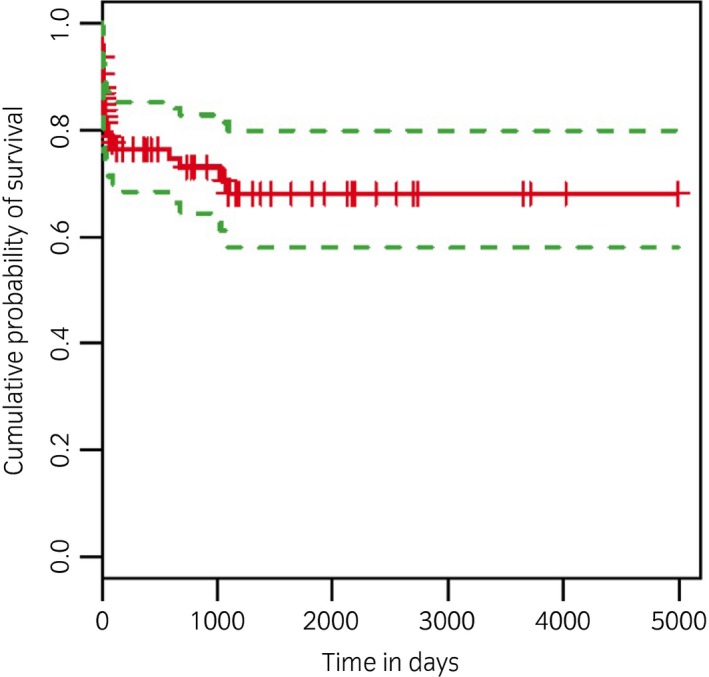
Kaplan–Meier plot of the cumulative probability of survival in 127 horses that underwent endoscopic treatment following calcaneal bursae sepsis. The green lines represent the upper and lower 95% confidence intervals. Vertical lines on the curve represent censoring times.

**Figure 3:**
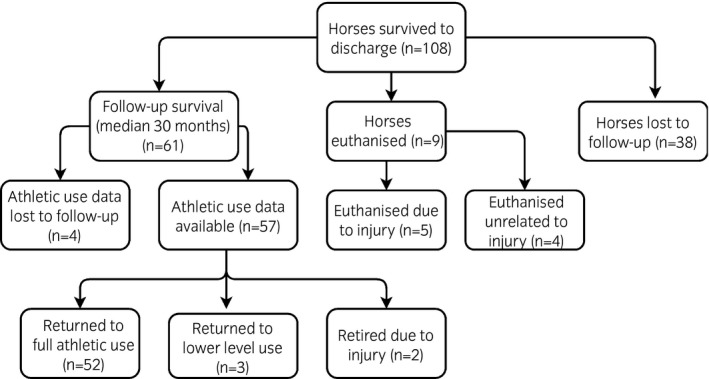
A flow chart to show progression of horses through the study following hospital discharge.

Of the 108 horses discharged from the hospital, follow‐up survival information was available for 70 (64.8%). Duration of follow‐up ranged from 3 months to 13 years, with a median of 30 months. Follow‐up revealed 87.1% (61/70) horses were still alive following hospital discharge, while 7.1% (5/70) had been euthanised due to the original injury and 5.7% (4/70) had been euthanised for unrelated reasons. The overall cumulative probability of survival for the horses in the cohort is shown in Figure 2. A flow chart to show progression of horses through the study following hospital discharge is shown in Figure 3. Data for return to athletic function was available for 57 horses with 91.2% (52/57) of horses having returned to same/higher level compared with before the injury; 55.8% (29/52) horses were competing at low level (unaffiliated), 30.8% (16/52) at intermediate level (affiliated national competition or hunting) and 13.5% (7/52) horses used for racing. Unaffiliated competitions are low level shows organised locally and not run under national rules. Affiliated competitions are run under national rules organised by a central organisation. Five horses did not return to the same/higher level of exercise (8.8%, 5/57) and of these three returned to a lower level of work (unaffiliated) while two had been retired because of persistent lameness of the injured limb.

### Univariable analysis

**Figure 4:**
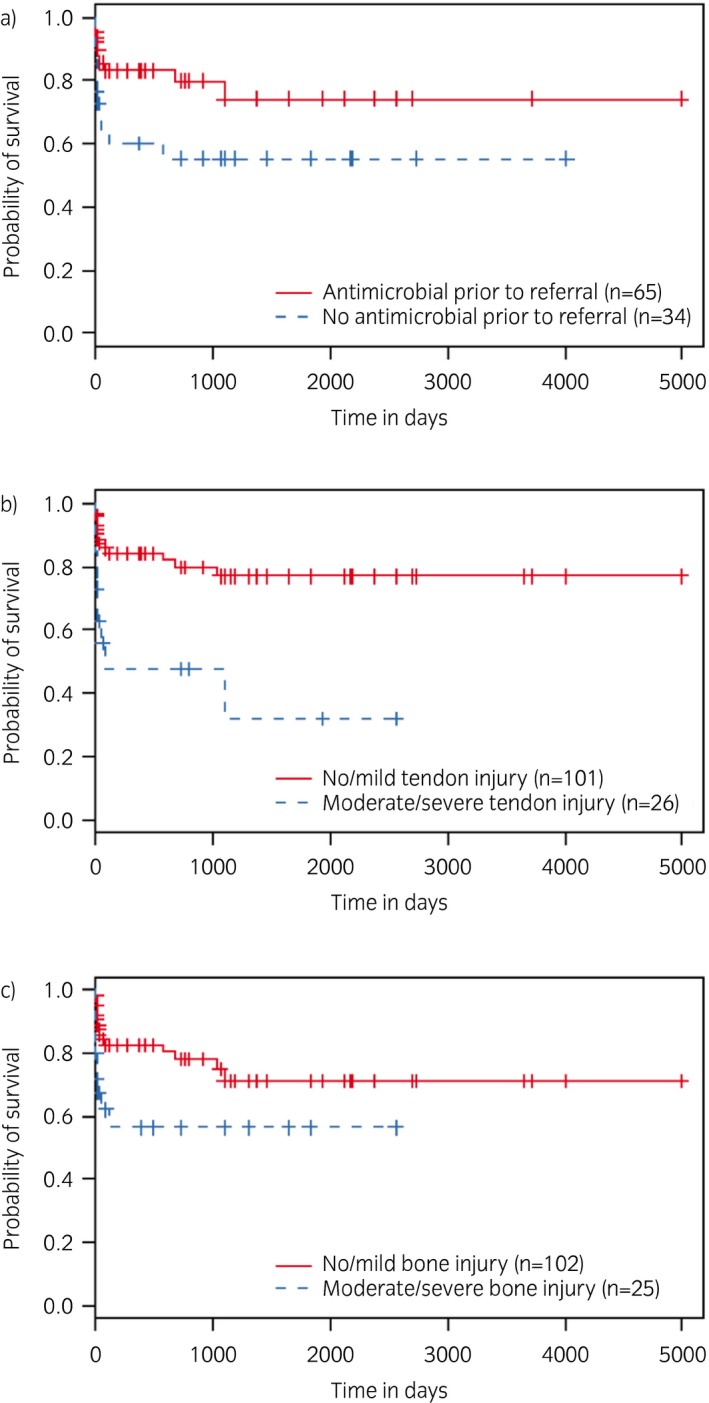
Kaplan–Meier plots of cumulative probability of survival and the log‐rank test were used to compare survival between horses where antimicrobials were or were not administered prior to referral a), horses with none/mild or moderate/severe tendon lesion b), and horses none/mild or moderate/severe bone involvement c). Vertical lines on the curves represent censoring times.

Univariable Cox proportional hazards analyses are available in Supplementary Item 1. In brief, the administration of systemic antimicrobials prior to referral (Hazard Ratio [HR] 0.41, 95% CI 0.18–0.91, P = 0.03) and time to referral was associated with reduced mortality (HR 0.89, 95% CI 0.80–0.99, P = 0.01) while moderate/severe tendon (HR 3.78, 95% CI 1.78–8.04, P = 0.001) and moderate/severe bone involvement (HR 2.43, 95% CI 1.12–5.26, P = 0.03), duration of general anaesthesia (HR 1.01, 95% CI 1.00–1.02, P = 0.04), post‐operative synoviocentesis (HR 3.18, 95% CI 1.36–7.43, P = 0.006) and post‐operative wound dehiscence (HR 2.50, 95% CI 1.08–5.65, P = 0.04) were associated with increased mortality. Kaplan–Meier plots illustrating the effect of administration of antimicrobials prior to referral and the presence of significant tendon and/or bone lesions on post‐operative survival are shown in Figure 4. None of the continuous variables considered for model building showed non‐linear association with the probability of survival and therefore all were included as a linear fit.

### Multivariable analysis

The results of the multivariable Cox proportional hazards model of variables associated with survival are shown in Table 1. The administration of systemic antimicrobials prior to referral was associated with reduced mortality (HR 0.25, 95% CI 0.11–0.60, P = 0.002) while moderate/severe tendon involvement (≥30% cross sectional area) was associated with increased mortality (HR 7.95, 95% CI 3.33–19.0, P<0.001). The proportional hazard assumption of the Cox proportional hazards model was met for both variables retained in the final model and there were no influential data points identified.

**Table 1:** Multivariable Cox proportional hazards model of variables associated with survival. The data are from 127 horses that underwent endoscopic surgery for treatment of septic calcaneal bursitis; only 98 horses were included in the model due to missing data for some variables

Variable	Coefficient	Standard error	Hazard ratio	95% CI of the hazard ratio	P value
Systemic antimicrobial treatment prior to referral (yes/no)	−1.39	0.44	0.25	0.11–0.60	0.002
Lesion associated with moderate/severe tendon injury (≥30% cross sectional area) (yes/no)	2.1	0.44	7.95	3.33–19.0	<0.001

CI, confidence interval.

## Discussion

This study revealed that endoscopic treatment of synovial contamination/sepsis of the calcaneal bursae has an 84% survival rate to hospital discharge. The study identified variables associated with survival, which include: early antimicrobial therapy was associated with a successful outcome and significant tendon damage was associated with treatment failure. Follow‐up survival was good with the majority of horses returning to previous athletic use at the same or higher level. This information can be used to identify horses at increased risk of non‐survival and therefore can be used to manage clients’ expectations regarding realistic outcomes following surgery.

The current study reported similar success rates compared with treatment of other contaminated or septic synovial structures in the horse 9, 17, 18 and a better success rate than previously reported for sepsis of calcaneal bursae 10. Reasons which may explain this improved success rate include the use of endoscopic treatment in all cases, improved knowledge of the anatomy 6, 19, 20 and aggressive and prudent use of both appropriate local and systemic antimicrobials 21. Early referral as well as administration of systemic antimicrobials prior to referral were associated with a more favourable outcome which highlights the importance of recognising the seriousness of the condition as early as possible.

Moderate/severe tendon injury was associated with decreased survival in the current study which is supported by previous equine studies 17, 18, 21, 22, 23. Bone damage was also a predictor of poor survival in the univariable analysis as reported in other publications 10, 18, 24, 25; however, this finding did not remain significant in the multivariable analysis. Possible reasons for this include low numbers of horses presented to the hospitals with moderate/severe bone injury, due to low frequency of bone involvement overall or failure of horses with this type of injury to be selected for surgical treatment. The influence of tendon and/or bone involvement emphasises the need for thorough radiographic and/or sonographic assessment for cases of calcaneal bursa contamination/sepsis. Furthermore, bone or tendon involvement may only become evident during endoscopic examination or following repeated radiography or ultrasonography at some time following the initial evaluation 26.

The reasons for treatment failure in cases with bone injury is likely due to persistent infection or instability 27. In cases with tendon injury, treatment failure is likely due to loss of function of important parts of the stay or suspensory apparatus 20. Both necrotic bone and tendon could become a source of persistent infection 28. Elimination of infected tissue is usually required to effectively treat these cases, therefore, loss of significant proportions of the bone integrity or tendon structure may further decrease the prognosis for athleticism 18, 29.

Systemic administration of antimicrobials prior to referral was associated with improved survival in the horses in our study, a similar finding from a previous study 21. Early initiation of antimicrobial therapy should be encouraged when evaluating and treating wounds to the calcaneal area. Gram‐positive bacteria were cultured most frequently at all of the referral hospitals included in this study, consistent with the findings from recent studies 21, 30. Gram‐negative bacteria were also cultured regularly, so despite the predominance of Gram‐positive bacteria, broad‐spectrum antimicrobials are recommended prior to referral unless bacteriological culture and susceptibility results are available, in accordance with antimicrobial stewardship 31. The current study did not identify any association between positive culture and negative outcome unlike previous studies 18, 32. A bacteriological sample was submitted in less than a third of all cases and it is plausible that the study cohort was too small to detect such an association. The majority of horses in the current study had received systemic antimicrobials prior to referral, which may have influenced the culture results.

Two horses in our study sustained a catastrophic fracture of the contralateral tibia during recovery from general anaesthesia, a higher incidence than the previously reported fracture prevalence (0.25%) 33. Neither mare had a cast for recovery nor underwent assisted recovery. For one mare the GA time was not recorded while for the other mare the duration of GA was 160 min. The mean GA time for this cohort was 122 min (interquartile range 100–140) so the mare with GA time data available underwent a prolonged duration of GA in relation to the study group. The overall broodmare population in this study was low (1.6%) but both horses that sustained fractures were aged mares and one was a pregnant broodmare. Age has been recognised previously as a risk factor of sustaining a fracture during recovery from anaesthesia 34 and a decrease in metacarpophalangeal break strength has been identified in mares post‐partum 35. Further studies are needed to investigate if horses with calcaneal bursa contamination/sepsis are at increased risk of contralateral limb fractures during recovery compared with horses undergoing general anaesthesia for other musculoskeletal injuries.

Limitations of the current study include incomplete records due to the retrospective nature, which limited the available data and therefore constraining/limiting the power of the statistical analysis. Diagnostic imaging findings and the degree of bone and tendon injury were obtained from clinical records and individual images were not reviewed by the authors. The follow‐up in the present study was opportunistic in some cases and therefore shorter than ideal. Despite the loss of 38 horses after hospital discharge (35%, 38/108), the overall median follow‐up time of 30 months falls in the reported long‐term follow‐up time frame (≥12 months) 36. The cohort in this multi‐centre study is a fair representation of the UK referral horse population; however, the results may not be relevant to different horse populations or geographical areas, in particular since horses for which referral was not an option are not included. The majority of the non‐survivors in the current study were due to client‐selected cessation of treatment leading to euthanasia. Euthanasia is not solely determined by disease pathology 37. As in this study, the owners’ decision for euthanasia was likely based on many factors including horse age and cost of treatment 38 and other factors relating to the disease, animal, owner and veterinary surgeon 37. Euthanasia is an arbitrary endpoint and is further complicated by losses of follow‐up and unknown causes of death 37, 38. Nevertheless, the difficulties associated with the application of survival analysis in veterinary epidemiology are inherent and euthanasia is in many situations the best available measure of treatment outcome 37, 39.

In conclusion, endoscopic treatment of calcaneal bursae contamination/sepsis in horses has an 84% survival rate to hospital discharge with the majority of horses returning to athletic function. This study is the first to report reduced survival with tendinous involvement in horses with calcaneal bursa contamination/sepsis and improved survival in horses that received systemic antimicrobials prior to referral. These findings assist identification of horses at increased risk of a poor outcome following treatment and should be used when advising owners on expected outcomes following surgery.

## Authors’ declaration of interest

No competing interests have been declared.

## Ethical animal research

Ethical approval for the study was granted by the University of Liverpool Veterinary Research Ethics Committee (VREC328).

## Owner informed consent

Owners gave consent for their animals' inclusion in the study.

## Sources of funding

The Horserace Betting Levy Board generously funds G. Lipreri and R.J.T.Y. Graham Clinical Scholarships.

## Authorship

C.M. Isgren, E.R. Singer and L.M. Rubio‐Martinez contributed to study design. C.M. Isgren, C.E. Wylie, G. Lipreri, R.J.T.Y., Graham contributed to study execution. S.E. Salem, C.M Isgren and C.E. Wylie, contributed to data analysis and interpretation. C.M. Isgren, S.E. Salem, E.R. Singer, C.E. Wylie, G. Lipreri, R.J.T.Y., Graham, B. Bladon, J.C. Boswell, A.R. Fiske‐Jackson, T.S. Mair and L.M. Rubio‐Martinez contributed to the preparation of the manuscript. All authors gave their final approval of the manuscript.

## Supporting information


**Supplementary item 1: **Categorical and continuous variables investigated for association with long‐term survival.

**Supplementary item 2**: Questionnaire used during telephone interview performed at least 3 months following hospital discharge.
