# Supplementary material for: A multi‐centre cohort study investigating the outcome of synovial contamination or sepsis of the calcaneal bursae in horses treated by endoscopic lavage and debridement
Source: Equine Vet J. 2019 Oct 16;52(3):404–10. doi: 10.1111/evj.13180 (PMC7186813; doi:10.1111/evj.13180)
Supplement: Supplementary file 1 — Supplementary item 1: Categorical and continuous variables investigated for association with long‐term survival. [file EVJ-52-404-s001.pdf]

BEVA Equine Veterinary Journal

Supplementary Item 1: Categorical and continuous variables investigated for association with long-term survival. Data were collected from 127 horses that underwent surgery for treatment of septic calcaneal bursitis and investigated for association with the risk of postoperative death using a univariable Cox proportional hazards model. LRT = likelihood ratio test; TB/TBX = Thoroughbred/Thoroughbred cross; WB/WBX = Warmblood/Warmblood Cross, IVRP = Intravenous Regional Perfusion; CB = Calcaneal bursa; ICB = Intertendinous calcaneal bursa; GCB = gastrocnemius calcaneal bursa; Ref. = reference category. Descriptive data are presented as numbers and percentages for categorical variables and median (interquartile range) for continuous variables.
*Model did not converge
Breed group 1: WB, WBX, TB, TBx, SB, Lusitano, Arab
Breed group 2: Pony cob draught

Variable	Category	Descriptive data, Number (%)	Coefficient	Standard error	Hazard ratio	95% confidence interval of the hazard ratio	LRT *p* value	% of missing data
I. Categorical variables								
Participating hospital	Hospital 1	19 (15)	Ref.					0.0
	Hospital 2	12 (9.4)	−0.39	0.83	0.68	0.13–3.5		
	Hospital 3	33 (26)	−0.05	0.57	0.95	0.31–2.9		
	Hospital 4	5 (3.9)	−0.19	1.1	0.82	0.1–7.1		
	Hospital 5	30 (23.6)	−0.12	0.63	0.89	0.25–3.1		
	Hospital 6	19 (15)	0.16	0.61	1.17	0.36–3.8		
	Hospital 7	9 (7.1)	−0.04	0.84	0.96	0.19–4.9	0.10	
Sex	Female	72 (58.1)	Ref.					2.4
	Male	52 (41.9)	0.24	0.39	1.27	0.6–2.72	0.54	
Breed	Arab	6 (5.1)	Ref.					7.1
	Cob/draught	10 (8.5)	−0.94	0.82	0.39	0.078–1.96		
	Other	2 (1.7)	1.4	0.92	4.06	0.66–24.82		
	Pony	23 (19.5)	−1.96	0.82	0.14	0.028–0.71		
	TB/TBX	65 (55.1)	−1.43	0.64	0.24	0.068–0.84		
	WB/WBX	12 (10.2)	−1.56	0.92	0.21	0.035–1.27	0.03	
Breed condensed	Group 1	85 (72)	Ref.					7.1
	Group 2	33 (28)	−0.33	0.46	0.71	0.29–1.76	0.45	
Affected leg	LH	61 (49.2)	Ref.					2.4
	RH	63 (50.8)	−0.49	0.38	0.61	0.29–1.3	0.14	
Lameness score at walk*	None	8 (7.3)						13.4
	Mild (1–3)	15 (13.6)						

Variable	Category	Descriptive data, Number (%)	Coefficient	Standard error	Hazard ratio	95% confidence interval of the hazard ratio	LRT *p* value	% of missing data
	Moderate (4-6)	43 (39.1)						
	Severe (7-10)	44 (40)						
Lameness score (categories condensed)	None/mild	23 (20.9)	Ref.					13.4
	Moderate/severe	87 (79.1)	0.9	0.74	2.47	0.58–10.6	0.17	
Open wound*	No	3 (2.4)						0.0
	Yes	124(97.6)						
Wound level	Proximal to POH	8 (6.8)	Ref.					7.1
	At POH	67 (56.8)	−1.01	0.57	0.33	0.12–1.02		
	Distal to POH	43 (36.4)	−1.26	0.61	0.28	0.08–0.94	0.16	
Wound location	Lateral	23 (20.4)	Ref.					11.0
	Medial	13 (11.5)	0.86	0.61	2.35	0.72–7.76		
	Multiple	8 (7.1)	−0.59	1.1	0.55	0.06–4.74		
	Plantar	69 (61.1)	0.04	0.52	1.03	0.37–2.89	0.33	
Synovial drainage from wound	No	50 (49.5)	Ref.					20.5
	Yes	51 (50.5)	0.16	0.44	1.17	0.44–2.77	0.72	
Systemic antimicrobial prior to referral	No	34 (34.3)	Ref.					22.0
	Yes	65 (65.7)	−0.89	0.41	0.41	0.18–0.91	0.03	
Local antimicrobial prior to referral	No	92 (96.8)	Ref.					25.2
	Yes	3 (3.2)	0.3	1.02	1.35	0.18–10.07	0.77	
Synovectomy performed during surgery	No	34 (29.1)	Ref.					7.9
	Yes	83 (70.9)	0.51	0.5	1.66	0.62–4.43	0.29	
Intrasynovial antimicrobial during surgery	No	20 (18.9)	Ref.					16.5
	Yes	86 (81.1)	−0.66	0.44	0.51	0.21–1.25	0.16	
Local antimicrobial during surgery (intrasynovial or IVRP)	No	5 (4.5)	Ref.					13.4
	Yes	105 (95.5)	−0.49	0.74	0.61	0.14–2.61	0.54	
Intrasynovial antimicrobial at the end of surgery	No	12 (11.1)	Ref.					15.0
	Yes	96 (88.9)	−0.83	0.5	0.43	0.16–1.16	0.13	
IVRP during hospitalisation	No	76 (66.1)	Ref.					9.5
	Yes	39 (33.9)	0.53	0.42	1.7	0.75–3.84	0.21	
Lesion involving superficial CB only*	No	115 (90.6)						0.0
	Yes	12 (9.4)						

Variable	Category	Descriptive data, Number (%)	Coefficient	Standard error	Hazard ratio	95% confidence interval of the hazard ratio	LRT *p* value	% of missing data
Lesion involving CB only*	No	66 (52.0)						0.0
	Yes	61 (48.0)						
Lesion involving both CB and SCB	No	73 (57.5)						0.0
	Yes	54 (42.5)						
Lesion associated with any degree bone injury	No	86 (74.1)	Ref.					8.7
	Yes	30 (25.9)	0.74	0.41	2.1	0.93–4.73	0.08	
Lesion with moderate/severe bone injury	No	101 (80.2)	Ref.					0.8
	Yes	25 (19.8)	0.89	0.4	2.43	1.12–5.26	0.03	
Lesion associated with any degree of tendon injury	No	52 (41.3)	Ref.					0.8
	Yes	74 (58.7)	0.61	0.42	1.84	0.81–4.18	0.13	
Lesion associated with moderate/severe tendon injury	No	100 (79.4)	Ref.					0.8
	Yes	26 (20.6)	1.33	0.38	3.78	1.78–8.04	0.001	
Primary wound closure achieved	No	19 (17.6)	Ref.					15.0
	Yes	89 (82.4)	−0.28	0.5	0.75	0.28–2.02	0.58	
Limb immobilised after surgery	No	93 (75.6)	Ref.					3.2
	Yes	30 (24.4)	0.71	0.41	2.04	0.91–4.55	0.09	
Cast placed within 24 hours of surgery	No	106 (86.2)	Ref.					3.2
	Yes	17 (13.8)	0.94	0.45	2.56	1.06–6.17	0.05	
Postoperative synoviocentesis	No	69 (60.5)	Ref.					10.3
	Yes	45 (39.5)	1.16	0.43	3.18	1.36–7.43	0.006	
Total number of bursoscopic lavage	One	106 (83.5)	Ref.					0.0
	More than one	21 (16.5)	0.71	0.42	2.03	0.9–4.62	0.09	
Postoperative wound dehiscence	No	77 (69.4)	Ref.					12.6
	Yes	34 (30.6)	0.9	0.42	2.50	1.08–5.65	0.04	
Lameness at walk on hospital discharge	No	82 (83.7)	Ref.					
	Yes	16 (16.3)	1.36	0.65	3.9	1.1–13.8	0.05	22.8
II. Continuous variables		Descriptive data Median (range)	Coefficient	Standard error	Hazard ratio	95% confidence interval of the hazard ratio	LRT p value	% missing data
Age (years)	-	9 (6–13)	0.01	0.03	1.01	0.94–1.09	0.73	5.50
Lameness score (0–10 scale)	-	5.5 (4–8)	0.14	0.08	1.15	0.99–1.33	0.06	13.4

Variable	Category	Descriptive data, Number (%)	Coefficient	Standard error	Hazard ratio	95% confidence interval of the hazard ratio	LRT *p* value	% of missing data
Time to referral (days)	-	1.5 (0–7)	-0.11	0.06	0.89	0.80–0.99	0.01	16.5
Duration of general anaesthesia (minute)	-	122 (100-140)	0.01	0.01	1.01	1.00–1.02	0.04	15.0
Duration of hospitalisation (days)	-	12 (8-15.5)	0.014	0.01	1.01	0.99–1.03	0.24	12.6
