# Supplementary material for: A multi‐centre cohort study investigating the outcome of synovial contamination or sepsis of the calcaneal bursae in horses treated by endoscopic lavage and debridement
Source: Equine Vet J. 2019 Oct 16;52(3):404–10. doi: 10.1111/evj.13180 (PMC7186813; doi:10.1111/evj.13180)
Supplement: Supplementary file 2 — Supplementary item 2: Questionnaire used during telephone interview performed at least 3 months following hospital discharge. [file EVJ-52-404-s002.pdf]

BEVA Equine Veterinary Journal

Supplementary Item 2: Questionnaire used during telephone interview performed at least 3 months following hospital discharge.

Phone Number (1):

Phone Number (2):

Calcaneal bursa follow-up Questionnaire

Name of owner: Name of Horse:

Date of surgery: Date of follow-up:

Introductory statement: I am telephoning with regards to XX, your horse that was treated by keyhole surgery for infection/ wound around hock region ("calcaneal bursa") at XX clinic. We are investigating the type of treatment required in these cases and the outcomes following surgery to try and help horses with the same injury in the future. Your participation is voluntary and you may withdraw consent at any time. All information is confidential.

Would you be prepared to answer a short questionnaire? **Yes / No**
*(if **No** interview is abandoned at this stage)*

Questions regarding survival

1. Is the horse still alive? **Yes / No**
2. If No: was euthanasia related to the hock injury/wound? **Yes / No**
3. Was euthanasia unrelated to hock injury/wound? **Yes / No**
4. Please specify the reason for euthanasia ______________________________
5. Date of euthanasia _________________________

Questions regarding post-operative care following discharge:

1. Did the horse require box rest following discharge from hospital? **Yes / No**
   -Less than 4 weeks
   - 5-8 weeks
   - Over 9 weeks
2. Did the horse require antimicrobials ("antibiotics") or NSAIDS ("painkiller") following discharge from hospital?
   Yes / No

- If possible can you remember the type?

3. Did the horse require any further surgery related to the hock injury/ wound?
   Yes / No

4. If there was a wound did the wound heal? **Yes/No**
   - How long did it take for the wound to heal?

5. Did the horse have the calcaneal bursa medicated? **Yes / No**

   - Corticosteroids/ hyaluronic acid/ IRAP/ PRP/ antibiotics/ unknown

6. Did the horse receive any complementary therapy? **Yes / No**

- Physiotherapy/ acupuncture/ other ___________________

Questions regarding return to athletic function

1. Did the horse regain full soundness on the affected limb following the hock injury/wound? **Yes / No**

   -Sound at walk? **Yes / No**
   -Sound at trot in a straight line? **Yes / No**
   -Sound when trotted on the lunge? **Yes / No**
   -Remained lame at walk? **Yes / No**
   -Remained lame at trot? **Yes / No**

2. Was soundness assessed by a veterinary surgeon? **Yes / No**

3. When did return to soundness occur:
-0-2 months
-2-4 months
-4-6 months
-6-9 months
-Greater than 9 months

7. Did the horse develop any complications as a result of the hock injury/wound?
   Yes / No
   If YES Osteoarthritis **Yes / No**
   Adhesions **Yes / No**
   Other______________________________

8. Has the horse subsequently developed any other lameness problems that have affected its ability to resume normal exercise? **Yes/ No**
   If YES which limb______________________
   Which limb was affected?____________________
   Cause of lameness if known_____________________

9. What level of work was the horse in prior to hock injury/wound?
   No work
   Leisure horse (light hacking/ schooling)
   Racing
   Affliliated competition horse: BE/ BSJA/ BD/ other

Low level/ unaffiliated competition horse
Other (please specify)______________________

10. To what level of work has the horse returned?
No work
Lower than previously
Same as previously
Higher than previously

11. If competition horse, has it returned to competing? **Yes / No**
